# R.B.N.A. Secretary's Preference for Many Words and Small Type

**Published:** 1919-03-01

**Authors:** 


### MORE ETHICS OF CONTROVERSY.

### R.B.N.A. Secretary's Preference for Many Words and Small Type.

THE following correspondence is instructive:

*Copy.*

THE HOSPITAL,
28 and 29 Southampton Street, Strand, W.C.2.
February 18, 1919.

MADAM,—As advised by telephone, your letter, owing to special pressure on our space at the time it reached us, is too long as it stands to ensure its publication in our columns this week. The substance of a substantial part of it has already appeared in THE HOSPITAL more than once. We have no hesitation, therefore, in inviting your kind co-operation to the extent of avoiding delay in publication by the omission of these repetitions, when we will do our best to publish the amended letter this week, providing it reaches our office by one o'clock to-morrow* (Wednesday).—We are, Yours faithfully,

(Signed) THE EDITOR.

To save you trouble we enclose a copy of your letter as it stands.

Miss Isabel Macdonald, Secretary R.B.N.A.

*[This invitation was not availed of.]

*Received by post February 20, 1919, after* THE HOSPITAL *of the 22nd instant was printed:*

*Copy.*

10 Orchard Street,
Portman Square, London, W. 1,
February 19, 1919.

